# Elevated Levels of Mislocalised, Constitutive Ras Signalling Can Drive Quiescence by Uncoupling Cell-Cycle Regulation from Metabolic Homeostasis

**DOI:** 10.3390/biom13111619

**Published:** 2023-11-06

**Authors:** Elliot Piper-Brown, Fiona Dresel, Eman Badr, Campbell W. Gourlay

**Affiliations:** Kent Fungal Group, School of Biosciences, University of Kent, Canterbury CT2 7NZ, UK

**Keywords:** yeast signalling, Ras, metabolism, yquiescence, cell fate

## Abstract

The small GTPase Ras plays an important role in connecting external and internal signalling cues to cell fate in eukaryotic cells. As such, the loss of RAS regulation, localisation, or expression level can drive changes in cell behaviour and fate. Post-translational modifications and expression levels are crucial to ensure Ras localisation, regulation, function, and cell fate, exemplified by RAS mutations and gene duplications that are common in many cancers. Here, we reveal that excessive production of yeast Ras2, in which the phosphorylation-regulated serine at position 225 is replaced with alanine or glutamate, leads to its mislocalisation and constitutive activation. Rather than inducing cell death, as has been widely reported to be a consequence of constitutive Ras2 signalling in yeast, the overexpression of *RAS2^S225A^* or *RAS2^S225E^* alleles leads to slow growth, a loss of respiration, reduced stress response, and a state of quiescence. These effects are mediated via cAMP/PKA signalling and transcriptional changes, suggesting that quiescence is promoted by an uncoupling of cell-cycle regulation from metabolic homeostasis. The quiescent cell fate induced by the overexpression of *RAS2^S225A^* or *RAS2^S225E^* could be rescued by the deletion of *CUP9*, a suppressor of the dipeptide transporter Ptr2, or the addition of peptone, implying that a loss of metabolic control, or a failure to pass a metabolic checkpoint, is central to this altered cell fate. Our data suggest that the combination of an increased *RAS2* copy number and a dominant active mutation that leads to its mislocalisation can result in growth arrest and add weight to the possibility that approaches to retarget RAS signalling could be employed to develop new therapies.

## 1. Introduction

The small guanosine triphosphate hydrolases (GTPases) of the Ras superfamily function as molecular switches within cells to convey extracellular stimuli to intracellular effectors. *S. cerevisiae* carries two Ras genes, *Ras1* and *Ras2*, which share significant homology with the Ras proteins found in mammals [1]. Indeed, yeast cells lacking endogenous Ras function can be rescued by the expression of mammalian H- or N-Ras [2]. This simple yeast has therefore been used to explore fundamental principles of Ras protein function in eukaryotes. In addition to its well-known role as an oncogene within mammalian systems [3], Ras proteins are important in regulating the virulence of several human and plant fungal pathogens [4]. Although no strategies to manipulate Ras proteins directly have emerged as therapeutic or antifungal options to date, this possibility remains enticing and an active area of research.

Post-translational modifications (PTMs) can regulate Ras’s subcellular localisation, affecting the interaction of Ras with specific classes of effectors and regulators [5]. Processing of the CAAX motif located at the C-terminal domain and the subsequent palmitoylation of Ras are vital for endomembrane and plasma membrane targeting [6,7]. Phosphorylation has also been shown to be important in the regulation of Ras’s localisation and may offer a new approach to targeting oncogenic Ras within cancer therapy [8]. In *S. cerevisiae*, the two Ras proteins, Ras1 and Ras2, have also been shown to rely on phosphorylation to direct localisation and activity [9], and several putative serine phosphorylation sites have been identified that may play important roles. For example in studies where Ras2 serine 214 was replaced with alanine, cells showed reduced glycogen accumulation and increased activity of the canonical downstream cAMP/PKA pathway that connects Ras signalling to cellular responses in yeast [9,10].

Ras proteins have been shown to localise to several compartments of the cell. For example, in *S. cerevisiae,* Ras2’s association with the ER has been shown to be modulated by the Ras inhibitor 1 (Eri1) [11,12]. Furthermore, Ras2 has been shown to accumulate at mitochondrial membranes [13]. Ras accumulation was observed at the mitochondrial compartment in strains lacking either *WHI2*, a phosphatase activator that is important in stress responses, [14] or *COX4*, which is essential for cytochrome c oxidase assembly and oxidative phosphorylation [15], where constitutive signalling promotes ROS production and cell death. Ras2 has also been reported to localise to the nucleus when grown in the presence of glucose [16]. In each case, changes in Ras2 localisation led to dramatic changes in downstream signalling and cell fate, suggesting that spatial and temporal control are crucial regulatory factors in yeast cells.

Here, we explore the outcomes of increasing the level of expression of Ras2 within the context of both its mislocalisation and inappropriate activation. We report that the introduction of mutations at serine position 225 that interfere with its phosphorylation status lead to its mislocalisation and the cAMP/PKA-dependent uncoupling of cell-cycle regulation from metabolic control when overexpressed. These effects could be rescued by the deletion of *CUP9*, which represses the dipeptide transporter *PTR2* and elevates the levels of a number of metabolite transporters, suggesting that Ras^ser225^-induced signalling effects are rooted in a loss of metabolic homeostasis. Our findings illustrate the importance of both appropriate expression levels and phosphorylation control of Ras2 within the coordination of cellular homeostasis and cell fate in *S. cerevisiae*. The finding that the mislocalisation of a constitutively active form of Ras can drive a quiescent state in yeast has implications for possible antifungal approaches that could target Ras activity outside of its “un-druggable” [17] GTPase regulatory domain.

## 2. Materials and Methods

### 2.1. Yeast Growth and Manipulation

All experiments were carried out using the wild-type BY4741 (*MATa hisΔ1 leu2Δ0 met15Δ0 ura3Δ0)* grown at 30 °C in either YPD or in defined minimal media (Formedium) lacking uracil or leucine to allow for plasmid selection and maintenance. The strain lacking *CUP9* (MATa his3Δ1 leu2Δ0 met15Δ0 ura3Δ0 Δcup9::KANMX) was obtained from the yeast deletion collection (Open Biosystems). Cell growth was measured using a BMG LABTECH SPECTROstar Nano automatic plate reader. The plasmid used to overexpress *PDE2* has been described previously [18].

### 2.2. Site-Directed Mutagenesis and Ras Expression Plasmids

The QuikChange Lightning Site-Directed Mutagenesis Kit (Agilent, Cheshire, UK) was used to perform mutagenesis of the RAS2 gene cloned within the Gateway entry plasmid PDONR221 RAS2 KAN (Addgene, Watertown, MA, USA). Primers were constructed using the QuikChange Primer Design software (http://www.genomics.agilent.com/primerDesignProgram.jsp) to introduce mutations encoding a change from serine position 225 of Ras2 to either alanine (Ras2S225A forward primer 5′-ggcattcacgacagttgtggcactgttcacatttttaccg-3′ and reverse primer 5′-cggtaaaaatgtgaacagtgccacaactgtcgtgaatgcc-3′) or glutamate (Ras2S225E forward primer 5′-cattcacgacagttgtctcactgttcacatttttaccgttggcagcattg-3′ and reverse primer 5′-caatgctgccaacggtaaaaatgtgaacagtgagacaactgtcgtgaatg-3′). The mutations were verified by Sanger sequencing. Low- and high-copy-expression plasmids expressing Ras2, Ras2S225A, or Ras2S225E were generated using a gateway LR cloning reaction (Thermo Fisher, Leicestershire, UK) using the destination vectors pAG416GPD-EGFP-ccdB and pAG426GPD-EGFP-ccdB.

### 2.3. GFP-Atg8 Autophagy Assay

An overnight culture was grown in defined minimal medium lacking uracil and containing 2% glucose at 30 °C with shaking at 200 rpm. The overnight culture was diluted to an OD_600_ of 0.1 in 5 mL of fresh minimal medium lacking uracil and containing 2% glucose. Cells were incubated at 30 °C with shaking at 200 rpm for 24 h. To induce autophagy via nitrogen starvation, cells were incubated at 30 °C with 200 rpm shaking for 6 h in nitrogen starvation medium (2% glucose, 0.675% yeast nitrogen base without amino acids and ammonium sulphate, 0.193% synthetic dropout media supplement lacking uracil, Formedium). Total protein was extracted from cells before and after the induction of autophagy and subjected to Western blotting using an anti-GFP antibody. 

### 2.4. Western Blotting

The primary antibody used for Ras2 detection, goat anti-Ras2 polyclonal IgG, was purchased from Santa Cruz Biotechnology (Santa Cruz Biotechnology, Inc. Bergheimer Str. 89-2, 69115 Heidelberg, Germany) and was used at a dilution of 1/1000. The secondary antibody for Ras2 detection was anti-sheep IgG HRP (Sigma, Hertfordshire, UK, catalogue number A3415) and was used at a dilution of 1/5000. The primary antibody used for the detection of Pgk1p, rabbit anti-Pgk1p, was a kind gift from Professor Mick Tuite of the University of Kent and was used at a 1/10,000 dilution. For the detection of GFP-Atg8, a mouse anti-GFP antibody (Sigma, 1181446000) at a dilution of 1/1000 was used, with secondary antibody detection with anti-mouse IgG HRP (Sigma, A4416) at a dilution of 1/5000. Where quantified for qualitative comparison, protein band intensities were assessed using the densitometry function of the open-source ImageJ platform (https://imagej.net/ij/), and the bands were normalised to the appropriate PGK band within each lane to take account of loading differences.

### 2.5. Chronological Ageing Assay

Cells were grown overnight in 5 mL of minimal medium lacking uracil and containing 2% glucose, and then they were sub-cultured to an OD_600_ of 0.1 in 10 mL of fresh minimal medium lacking uracil and containing 2% glucose in 100 mL conical flasks. The samples were incubated at 30 °C, with shaking at 200 rpm. 

After 24 h of incubation, an aliquot of the culture was taken to calculate the colony-forming units and for flow cytometry analysis to measure the levels of propidium iodide (necrotic cells) and dihydroethidium fluorescence (ROS). Aliquots of cells were taken from the same culture sequentially at 24 h intervals for 6 days, and colony-forming unit counts and flow cytometry analysis were performed. The final measurement was taken after 12 days of sample incubation. To check for the presence of necrotic cells, propidium iodide was added at a concentration of 0.4 µM for 30 s before being placed into a BD Accuri flow cytometer and assessed for excitation at 535 nm and emission at 617 nm. To assess ROS levels, the dihydroethidium (DHE) dye (Invitrogen, Waltham, MA, USA, D23107) was used for the detection of superoxide radicals; 2 × 10^6^ cells were resuspended in PBS containing 10 μM DHE and incubated for 15 min before detection with a BD Accuri flow cytometer (500 nm and an emission peak at 582 nm). For the assessment of PI and DHE staining, a baseline was set using an unlabelled control, and all experiments were repeated in biological triplicate.

### 2.6. CFU and Viability Assays

Cells from an overnight culture were diluted to an OD_600_ of 0.1 and incubated at 30 °C for 24 h. The cells were then diluted to 2 × 10^3^ cells/mL, and 300 cells were plated onto the appropriate medium in technical and biological triplicates. The percentage viability was determined by the number of arising colonies. 

### 2.7. Fluorescence Microscopy

For the visualisation of GFP fluorescent cells (excitation/emission 488/512 nm), an Olympus 1X81 inverted microscope with a Cool LED pE4000 illumination system and an Andor Zyla 4.2 PLUS sCMOS camera was used. The acquisition software used to obtain the images was Micro-Manger, version 1.4.22. The experiments were conducted in biological triplicate, and representative images are presented. 

### 2.8. High-Resolution Respirometry

The Oroboros O2K Oxygraph High-Resolution Respirometer was used to determine the consumption of O_2_ in the cell suspensions. Datlab 4 software was used for the analysis and acquisition of data generated by the respirometer. Cell suspensions were diluted to a cell density of 10^6^ cells/mL and a routine respiration value (routine) was obtained, followed by the administration of the following respiratory chain inhibitors, in order: 0.2 mM triethiltyn bromide (TET, Sigma-Aldrich, UK), a complex V inhibitor used to assess respiration levels not linked to proton movement across the ATP synthase, referred to as LEAK respiration; 12 µM carbonylcyanidep-trifluoromethoxyphenylhydrazone (FCCP, Fluka, UK), a proton ionophore that reveals maximum (or uncoupled) respiration, referred to as ETS respiration; 2 µM antimycin A (AntA, Sigma-Aldrich), a complex III inhibitor that shows NMT or non-mitochondrial respiration. All experiments were performed in biological triplicate, as indicated.

### 2.9. RNA Sequencing and Gene Set Enrichment Analysis

Total RNA was extracted from 5 × 10^7^ yeast cells grown in biological triplicate at 30 °C to log phase in SD-LEU medium containing 2% glucose using the E.Z.N.A. Yeast RNA Kit Spin protocol (Omega Bio-tek, London, UK). An Illumina NextSeq 500 platform was utilised, producing 75 bp single-end reads (Novogene, Cambridge, UK). For each library, 20–30 M reads were generated. The analysis of RNA-Seq samples was performed using the Galaxy web platform (www.usegalaxy.org). The quality of the RNA sequencing reads was checked using FastQC v0.11.5 with default settings. Low-quality ends (Phred score < 20) and any adaptor sequences were trimmed using TrimGalore! v0.4.3. Reads shorter than 40 bp after trimming were not progressed for further analysis. After trimming, the quality was checked again using FastQC v0.11.5 to ensure correct trimming. Processed reads were aligned with the reference *S. cerevisiae* genome S288C version R64-2-1_20150113 (SacCer3) using HISAT2 v2.1.0 with single-end reads and reverse-strand settings. After alignment, the number of mapped reads was determined using the featureCounts plugin v1.6.3 with default settings. Reads aligning to multiple positions or overlapping more than one gene were discarded, counting only reads that mapped unambiguously to a single gene. Differential gene expression analysis between conditions was performed using DESeq2 v1.18.1 with default settings. To identify the cellular pathways containing differentially expressed genes, we conducted gene set enrichment analysis (GSEA; Broad Institute, Cambridge, MA, USA) [19]. To conduct GSEA, a list of significantly (q ≤ 0.05) differentially expressed genes, ranked from the most upregulated to the most downregulated, was uploaded into the GSEA database and compared to a gene set database (S288C version R64-2-1_20150113_features gtf annotation file).

## 3. Results

We wished to determine novel phosphorylation events that may influence Ras2’s localisation in *S. cerevisiae*. As a starting point, we made use of the PhosphoPep version 2.0 project database (www.phosphopep.org/index.php) [20]. This database allows users to search mass spectrometry data that compare the presence and levels of phosphorylated peptides between the proteomes of wild-type and gene-knockout yeast strains. The use of this database also allows for the prediction of regulatory kinases and phosphatases based on known consensus sequences. Using Ras2 as the search term and default parameters, the database’s output suggested that the loss of several enzymes led to changes in Ras2-derived phosphorylated peptides. The loss of kinases Mck1, Cka1, Ypk1, Pkp1, Bud32, Ssn33, Ctk1, and Pho85 or phosphatases Psr1, Psr2, and Sit4 led to changes in phosphorylated Ras2 peptide levels (Table S1). Upon examination of Ras2’s localisation in strains deleted for each enzyme predicted to change Ras’s phosphorylation status, only cells lacking *SSN3*, *SIT4*, or *PHO85* led to changes in GFP-Ras2 localisation (Supplementary Figure S1). Interestingly, the loss of either *SSN3* or *SIT4* led to an increase in the number of Ras2 peptides phosphorylated at the same residue: serine 225 (Table S1). To investigate whether the phosphorylation of Ras2 at serine 225 is important for its localisation, we replaced the amino acid with either glutamate (*RAS2^S225E^*, phospho-mimic) or alanine (*RAS2^S225A^*, non-phosphorylatable) via site-directed mutagenesis. We also cloned *RAS2*, *RAS2^S225A^*, and *RAS2^S225E^* into both low- and high-copy-expression plasmids driven by a constitutive GPD promoter, to investigate the effects of gene expression levels, and introduced these into wild-type cells.

**Figure 1:**
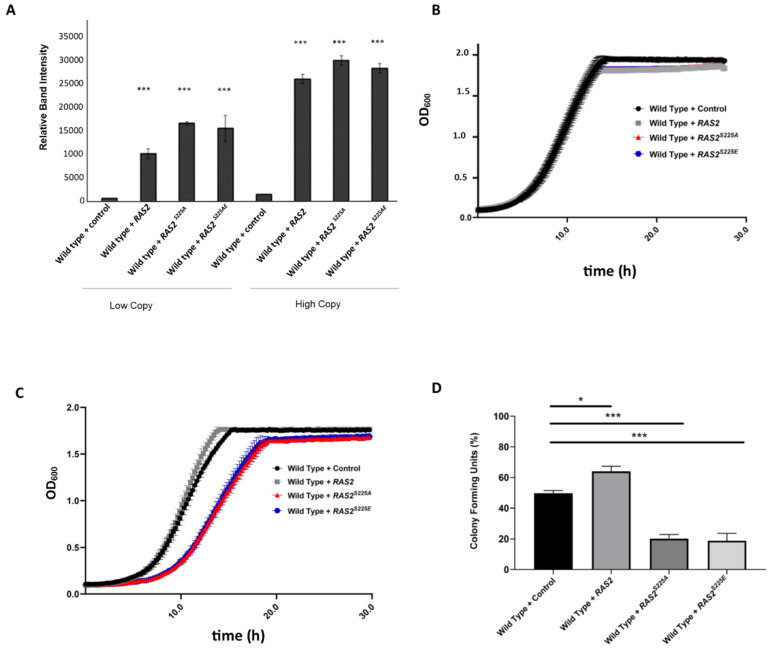
Cells expressing *RAS2*, *RAS2^S225A^*, and *RAS2^S225E^* from either a low-copy (CEN) or high-copy (2 µ) plasmid were grown in selective SD-URA minimal medium for 24 h at 30 °C before the total protein was extracted and probed by Western blotting with an anti-Ras2 and anti-Pgk1 antibody (loading control). Graphs represent the normalised relative band intensity from three biological replicates (**A**). Growth analysis of wild-type *S. cerevisiae* cells overexpressing *RAS2*, *RAS2^S225A^*, *RAS2^S225E^*, or an empty plasmid control (EV) from either a low-copy (CEN) (**B**) or high-copy (2 µ) (**C**) plasmid, representing an average of three biological replicates. Colony-forming unit assay of cells overexpressing *RAS2*, *RAS2^S225A^*, *RAS2^S225E^*, or an empty plasmid control grown in SD-URA medium (**D**). A one-way ANOVA using Dunnett’s multiple comparison test was used to determine statistical significance; * *p* ≤ 0.05, *** *p* ≤ 0.001. Error bars represent standard deviations.

To validate the expression levels of *RAS2*, *RAS2^S225A^*, and *RAS2^S225E^*, we conducted Western blots (Figure 1A, Supplementary Figures S2 and S3). Low-copy plasmids led to a tenfold increase in the expression level of *RAS2* and a fifteenfold increase in the levels of *RAS2^S225A^* and *RAS2^S225E^* (Figure 1A). The expression level was further increased to twenty-fivefold in strains expressing *RAS2*, *RAS2^S225A^*, and *RAS2^S225E^* from a multi-copy plasmid (Figure 1A). Despite the high levels of expression of *RAS2*, *RAS2^S225A^*, or *RAS2^S225E^* from a low-copy plasmid, we did not observe any changes in growth (Figure 1B). In contrast, the further increase in expression observed from the introduction of a high-copy plasmid led to a significant reduction in growth, but only in cells expressing *RAS2^S225A^* or *RAS2^S225E^* (Figure 1C). The viability of wild-type cells containing an empty high-copy plasmid grown to the stationary phase in the minimal selective medium used was around 50% when assayed at 24 h (Figure 1D), which represents the stationary phase of growth (Figure 1C). The overexpression of *RAS2* led to a significant increase in viability when compared to the wild-type control; however, viability was significantly reduced by the overexpression of *RAS2^S225A^* or *RAS2^S225E^* (Figure 1D). These data suggest that the modification of Ras2 at S225 can have a dominant negative impact on viability, but that the expression levels need to exceed a regulatory threshold to achieve this. 

**Figure 2:**
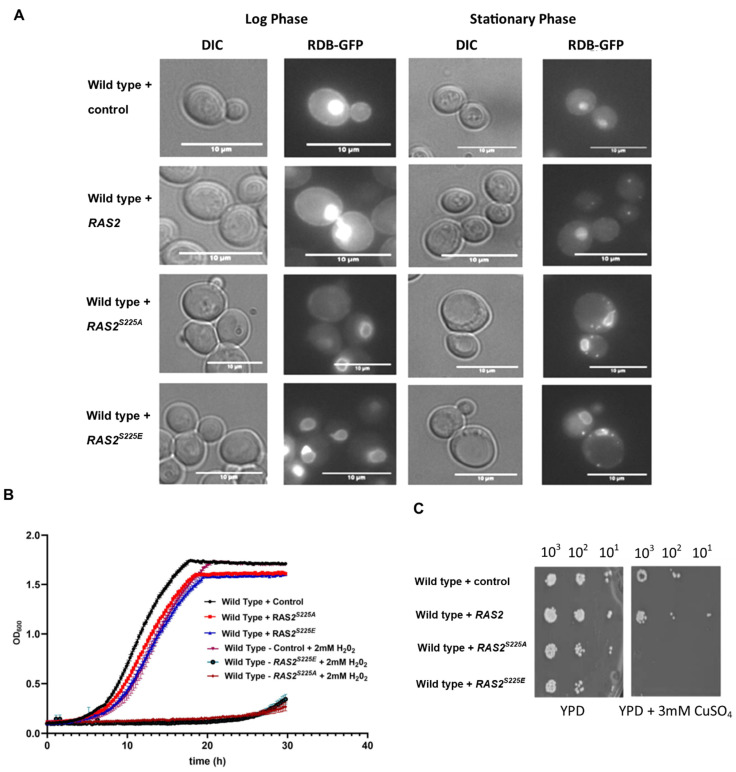
Active Ras was visualised in cells overexpressing *RAS2*, *RAS2^S225A^*, *RAS2^S225E^*, or a control containing an empty plasmid using a 3xGFP-RBD probe during the logarithmic and stationary phases of growth. Cells were cultured in SD-URA/-LEU growth media. The experiment was repeated three times, and a representative dataset is shown. Scale bar—10 µm (**A**). Growth of wild-type cells overexpressing *RAS2*, *RAS2^S225A^*, *RAS2^S225E^*, or containing an empty plasmid control was carried out in SD-URA or SD-URA + 2 mM H_2_O_2_ media; n = 3, error bars represent the standard deviation (**B**). Wild-type cells overexpressing *RAS2*, *RAS2^S225A^*, *RAS2^S225E^*, or containing an empty plasmid control were serially diluted from 2 × 10^6^/mL to 2 × 10^3^/mL and plated onto SD-URA plates supplemented with increasing concentrations of copper sulphate. This experiment was completed three times, and a representative result is shown (**C**).

To investigate whether the expression of *RAS2^S225A^* or *RAS2^S225E^* influenced Ras2’s localisation and activity, we made use of a construct that expresses the Ras-binding domain of human Raf1 fused to GFP [14]. As the RBD domain binds to Ras in its active GTP-bound state, this probe can give an account of the localisation of active Ras in living cells. The localisation of active Ras was analysed during the logarithmic and stationary phases of growth in cells overexpressing *RAS2*, *RAS2^S225A^*, and *RAS2^S225E^* from a high-copy plasmid (Figure 2A). During logarithmic growth, the wild-type controls and those overexpressing Ras2 showed localisation of GFP-RBD and, thus, active Ras at the plasma membrane and within the nucleus, as has been previously reported [14] (Figure 2A). In contrast, cells expressing *RAS2^S225A^* or *RAS2^S225E^* showed a strong GFP-RBD signal at the nuclear envelope (Figure 2A). During the stationary phase the RBD-GFP probe signal was seen as a diffuse cytoplasmic signal in wild-type and Ras2-overexpressing cells, as has been previously reported, reflecting the reduction in Ras activity during this phase of growth [14] (Figure 2A). In contrast, wild-type cells overexpressing either *RAS2^S225A^* or *RAS2^S225E^* maintained the nuclear envelope GFP-RBD signal, indicating the sustained localisation of active Ras at this location (Figure 2A). Intracellular foci were also observed in *RAS2^S225A^*- and *RAS2^S225E^*-expressing strains within the stationary phase (Figure 2A). These data suggest that the overexpression of *RAS2^S225A^* or *RAS2^S225E^* leads to constitutive activation, most notably at the nuclear envelope, throughout growth. 

The constitutive activation of Ras2 is often associated with a reduced ability to respond to oxidative stress. To test this, we grew strains in the presence of either hydrogen peroxide or copper sulphate, both of which led to a significant inhibition of growth in *RAS2^S225A^*- and *RAS2^S225E^*-expressing strains when compared to wild-type or *RAS2*-overexpressing strains (Figure 2B,C). 

### 3.1. Overexpression of RAS2^S225A/E^ Promotes a Quiescent Cell Fate

**Figure 3:**
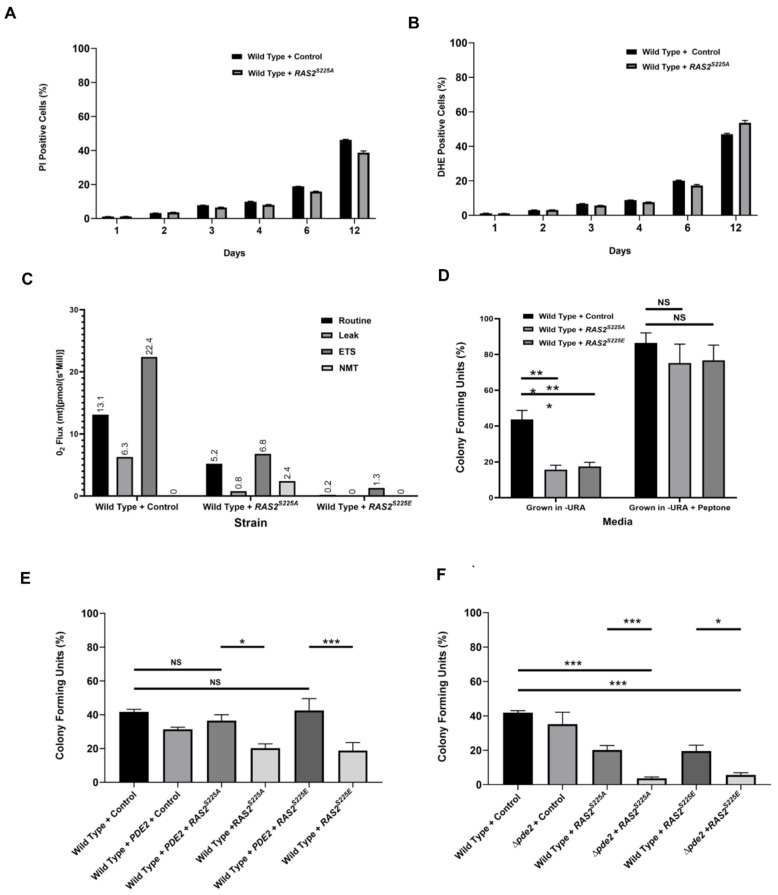
Wild-type cells overexpressing *RAS2^S225A^* or an empty plasmid control were grown in SD-URA, and necrosis (PI uptake) (**A**) and ROS (DHE) (**B**) measurements were taken over a 12-day period of continuous incubation. The data displayed are the average of three technical repeats, and the error bars represent the standard deviation. A bar chart showing the routine, leak, ETS, and NMT O_2_ flux values for wild-type cells overexpressing *RAS2^S225A^* or *RAS2^S225E^*, or containing an empty plasmid backbone control. The experiment was conducted in triplicate, and a representative dataset from one experiment is shown (**C**). A colony-forming unit assay of wild-type cells overexpressing *RAS2^S225A^* or *RAS2^S225E^*, or containing an empty plasmid control, grown in SD-URA medium for 24 h at 30 °C, was conducted and plated on either SD-URA or SD-URA + peptone (**D**). A colony-forming efficiency assay of wild-type cells overexpressing *RAS2^S225A^*, *RAS2^S225E^*, or an empty plasmid backbone co-expressed with *PDE2* (**E**), or in a strain lacking *PDE2* (**F**). The data presented are the average of three biological replicates, and the error bars represent the standard deviation. A one-way ANOVA using Tukey’s multiple comparison test was used to determine statistical significance. Nonsignificant = NS, * = adjusted *p*-value ≤ 0.01, ** adjusted *p*-value of 0.05 and *** = adjusted *p* value ≤ 0.001.

Changes in Ras’s localisation and activity often lead to an alternate cell fate. We therefore sought to determine the cell fate associated with the loss of viability observed upon overexpression of *RAS2^S225A^* or *RAS2^S225E^*. Loss of viability within a culture can occur as a result of cell death, which can be determined by the presence of cell-death-associated markers such as high levels of reactive oxygen species (ROS) or a loss of plasma membrane integrity (necrosis), as assessed by the uptake of the impermeant dye propidium iodide. The presence of high ROS containing and necrotic cells increases in a culture that is left to grow and deplete nutrients from the medium over time; this is commonly referred to as chronological ageing. Despite the overexpression of *RAS2^S225A^* or *RAS2^S225E^* leading to an increase in sensitivity to oxidative stress and exhibiting a loss of viability, we did not observe an increase in ROS production or propidium iodide uptake during chronological ageing when compared to the wild-type (Figure 3A,B). In addition, autophagy, a key process that is activated upon nutrient depletion and necessary for survival during chronological ageing, remained fully responsive, as assessed by the hydrolysis of GTP-ATG8, when *RAS2, RAS2^S225A^*, or *RAS2^S225E^* was overexpressed (Supplementary Figure S4). However, the loss of viability associated with *RAS2^S225A^* or *RAS2^S225E^* overexpression was linked to a dramatic reduction in mitochondrial respiration when assayed at 24 h of growth (Figure 3C). 

A lack of cell death markers and reduced respiration profile led us to investigate whether the loss of viability associated with *RAS2^S225A^* or *RAS2^S225E^* overexpression was in fact a consequence of quiescence. This assumption was supported by the fact that *RAS2^S225A^*- or *RAS2^S225E^*-overexpressing cells grown to 24 h in selective minimal medium could be revived by plating them onto an alternative rich medium (YPD) (Figure 3D). The cell viability was also increased when wild-type control cells were plated onto rich YPD medium (Figure 3D). From this, we hypothesised that nutritional limitation, bestowed by the selective media used in our experiments, led to a quiescent state in a proportion of wild-type cells, which was further exacerbated by the overexpression of *RAS2^S225A^* or *RAS2^S225E^*. Our findings also suggest that Ras2’s localisation and signalling play important roles in mediating the metabolic plasticity required for growth under minimal medium conditions. Ras2 mediates its signalling by controlling the activity of adenylate cyclase (Cyr1), which generates cAMP, which, in turn, activates protein kinase A (PKA). Activated PKA then phosphorylates a number of downstream targets that control a range of cellular responses. The increase in quiescence observed with the overexpression of *RAS2^S225A^* or *RAS2^S225E^* could be restored to wild-type levels by overexpressing the high affinity cAMP phosphodiesterase, *PDE2*, which reduces cAMP levels and PKA signalling (Figure 3E). In addition, the deletion of *PDE2*, which elevates cAMP levels, led to a further reduction in viability in cells expressing *RAS2^S225A^* or *RAS2^S225E^* (Figure 3F). These findings suggest that the overexpression of *RAS2^S225A^* or *RAS2^S225E^* promotes quiescence via cAMP/PKA signalling. 

### 3.2. RAS2^S225A/E^ Overexpression Uncouples Cell-Cycle Regulation from Metabolic Homeostasis

**Figure 4:**
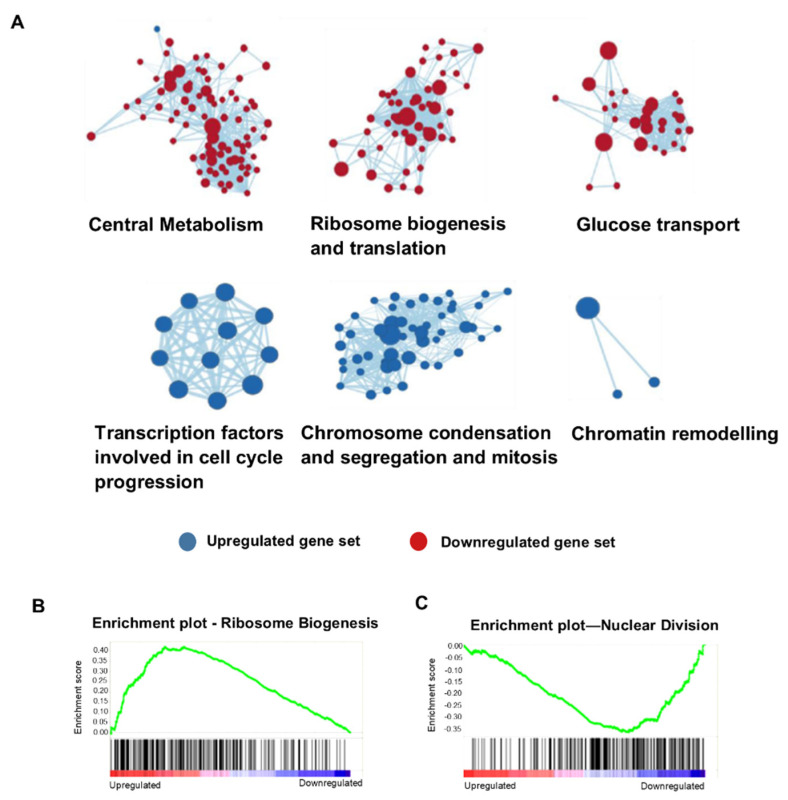
(**A**) Global gene expression changes in wild-type cells overexpressing *RAS2^S225A^* when compared to a wild-type control. DESeq2 was used to compare gene expression in wild-type cells overexpressing RAS2S225A to a wild-type control; in total, there were 4133 significantly differentially expressed genes. Gene set cluster maps were created using the Cytoscape plugin, showing the most upregulated and downregulated gene sets, as determined by GSEA analysis, along with their cellular functions; circle size within a cluster represents the change in the expression level of a single gene. Representative gene sets shown to be upregulated (**B**) or downregulated (**C**) upon the overexpression of *RAS2^S225A^* when compared to the wild-type control by GSEA. Vertical black lines represent individual genes in the significantly differentially expressed ranked gene list, from upregulated (**left**) to downregulated (**right**). An increase in the enrichment score is seen if there are many genes towards the beginning of the ranked list (upregulated) in the gene set.

We conducted RNA sequencing to help determine the cellular state underpinning *RAS2^S225A^*- and *RAS2^S225E^*-induced quiescence. To identify the cellular pathways with which differentially expressed genes were associated, we conducted a gene set enrichment analysis (GSEA; Broad Institute) [19]. Significantly (q ≤ 0.05) differentially expressed genes, ranked from the most upregulated to the most downregulated, from *RAS2^S225A^*- or *RAS2^S225E^*-expressing cells were compared to wild-type controls and to the GSEA database. GSEA functions to identify whether the members of each gene set are randomly distributed or significantly overrepresented (enriched) within a Gene Ontology term. using a normalised enrichment score (NES). Our data showed that overexpression of either *RAS2^S225A^* or *RAS2^S225E^* led to changes in precisely the same gene sets. We observed significant upregulation in pathways involved in growth, including ribosomal biogenesis, transcription, translation, glucose transport, and central metabolism—hallmarks of actively dividing cells (Figure 4A,B). However, we also observed a significant downregulation in GSEA gene sets in *RAS2^S225A^*- and *RAS2^S225E^*-expressing cells linked to cell growth, including cytoskeleton, microtubule, and kinetochore dynamics, nuclear division, chromosome condensation and segregation, cell-cycle phase transition, mitosis, and transcription factors associated with cell-cycle phase progression. (Figure 4A,C). These data suggest that the overexpression of *RAS2^S225A^* or *RAS2^S225E^* leads to a loss of coordination between processes that are essential for growth and offers a reasonable explanation as to the observed increase in the loss of viability associated with quiescence.

### 3.3. Loss of the Repressor Cup9 Rescues the Effects of Ras2^ser225A/E^ Overexpression

**Figure 5:**
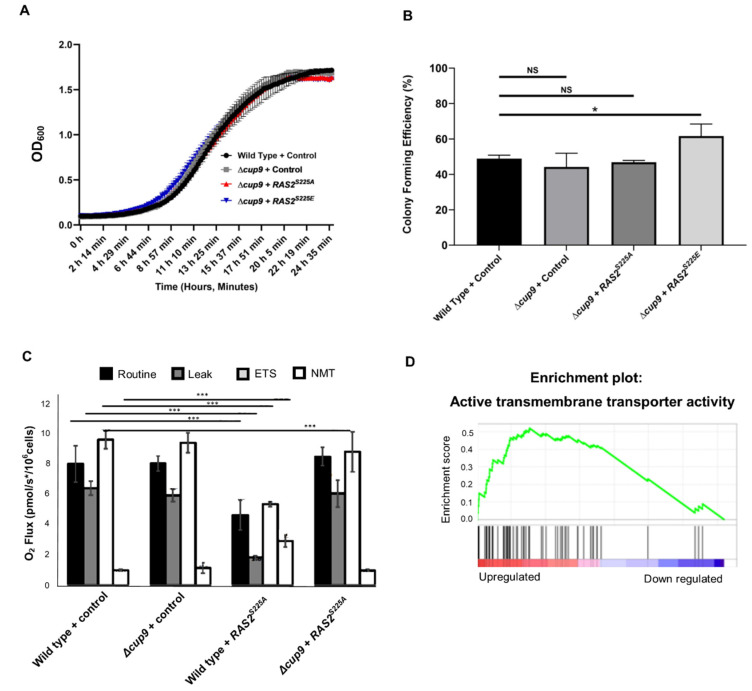
Growth analysis (**A**) and CFU assay (**B**) of wild-type and *Δcup9* cells overexpressing *RAS2^S225A^* or *RAS2^S225E^*, or containing an empty plasmid control. Routine, leak, ETS, and NMT O2 flux values for wild-type and *Δcup9* cells overexpressing *RAS2^S225A^* or an empty plasmid backbone control. In each case, the data shown represent an average of three biological repeats, and the error bars indicate the standard deviation. A one-way ANOVA using Tukey’s multiple comparison test was used to determine statistical significance; Nonsignificant = NS, * *p* ≤ 0.01, *** *p* ≤ 0.001 (**C**). A representative GSEA gene set shown to be upregulated upon the overexpression of *RAS2^S225A^* in a *Δcup9* background when compared to wild-type cells overexpressing *RAS2^S225A^* (**D**).

Given that the addition of a rich nutrient source was able to reactivate quiescent cells overexpressing *RAS2^S225A^* or *RAS2^S225E^*, we wished to determine the nutritional queue(s) responsible. The addition of additional glucose did not result in the relief of quiescence when *RAS2^S225A^* or *RAS2^S225E^* cells grown overnight were re-inoculated into fresh media (Supplementary Figure S5). As di/tripeptides are among the principal components of rich yeast growth media, we tested whether deletion of the repressor Cup9, which increases expression of the di/tripeptide transporter Ptr2, would allow *RAS2^S225A^* or *RAS2^S225E^* cells to re-enter cell growth when placed onto fresh minimal medium. The deletion of *CUP9* restored growth in cells overexpressing *RAS2^S225A^* or *RAS2^S225E^* to wild-type levels (Figure 5A). Viability was also restored in cells overexpressing *RAS2^S225A^*, with a slight increase in cells overexpressing *RAS2^S225E^* when *CUP9* was deleted when compared to the wild-type (Figure 5B). As the overexpression of *RAS2^S225A^* or *RAS2^S225E^* led to essentially the same phenotypes, we proceeded with further detailed analysis of metabolism using only *RAS2^S225A^*-overexpressing cells. The loss of *CUP9* alone did not affect respiration (Figure 5C). However, in line with the reversal of quiescence, the loss of respiration observed in *RAS2^S225A^*-overexpressing cells was reversed upon *CUP9*’s deletion (Figure 5C). To determine the nature of the metabolic changes that underpin this rescue, we compared the transcriptomes of *RAS2^S225A^* and *Δcup9 RAS2^S225A^* cells by GSEA. The significantly (q ≤ 0.05) differentially expressed genes were ranked from the most upregulated to the most downregulated and analysed by gene set enrichment analysis. Of the 202 genes that were significantly upregulated comparing *RAS2^S225A^* and *Δcup9 RAS2^S225A^* cells, we observed that in addition to the well-known target of Cup9 repression, *PTR2*, 40 genes were clustered within the term of transmembrane transporters. These included a number of genes involved in amino acid transport (*BAP2*, *BAP3*, *TAT1*, *ATG22*, *MMP1*, *DIP5*, and SAM3) and metal ion uptake (*FRE1*, *FRE7*, *CCC1*, *YOR1*, *PIC2*, and *ARN1*) (Figure 5D, Supplementary Table S2). A broader but uncharacterised role for *CUP9* in regulating metabolism was observed when RNA sequencing data from wild-type and *Δcup9* cells were compared using Gene Ontology within the yeastmine database. This highlighted that genes involved in the processes of pyruvate metabolism, glycolysis, carbohydrate catabolism, organic acid metabolism, and cytoplasmic translation were significantly enriched upon the deletion of *CUP9* (Supplementary Table S3). The deletion of *CUP9* also led to a significant downregulation of genes involved in rRNA modification, with a focus on methylation and sulphur metabolism (Supplementary Table S4).

**Figure 6:**
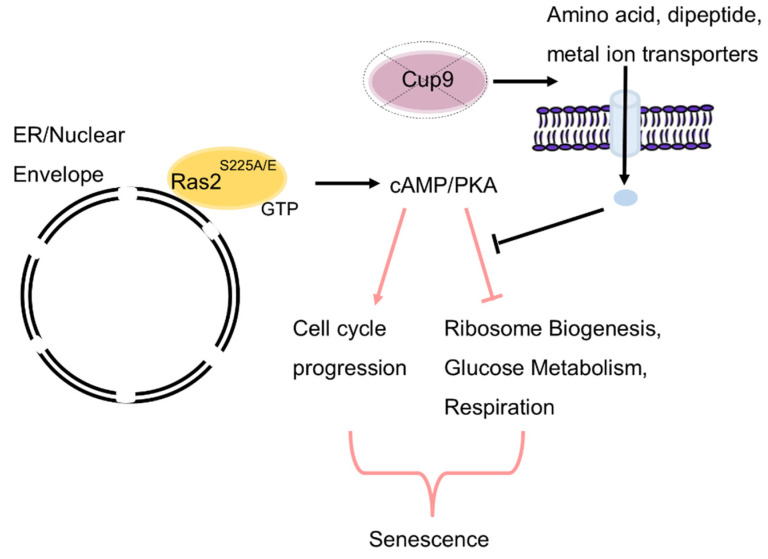
Model depicting a mechanism by which the overexpression of *RAS2^S225A^* or *RAS2^S225E^* promotes quiescence. The substitution of Ras2 at serine 225 for alanine or glutamate leads to constitutive activation at the nuclear envelope/ER when overexpressed. *RAS2^S225A/E^*-driven cAMP/PKA signalling from the nuclear envelope/ER, in turn, promotes senescence under conditions of nutritional challenge by uncoupling the control of the expression of cell-cycle control for core metabolic processes. The addition of peptone or deletion of *CUP9*, which leads to the upregulation of a battery of metabolite transporters, can counteract quiescence driven by *RAS2^S225A/E^* signalling, potentially by overcoming an essential metabolic checkpoint that is required to re-enter the cell cycle.

Overall, our data suggest that the overexpression of *RAS2^S225A^* or *RAS2^S225E^* alleles results in a quiescent state that is driven by changes in metabolism that do not support entry into or maintenance of an active cell cycle within a minimal nutrition environment. The deletion of *CUP9* appears to de-repress genes involved in the transport of metabolites, facilitating escape from this quiescent state (Figure 6). We also highlight a previously uncharacterised role for Cup9 in the control of genes involved in a range of metabolic processes required for normal growth.

## 4. Discussion

The correct regulation of Ras/cAMP/PKA activity is crucial for the integration of cell growth, cell-cycle progression, and metabolic activity [21,22,23,24]. As an example, aberrant activation of the Ras/cAMP/PKA pathway can lead to an incorrect diauxic reprogramming of the cell during nutrient depletion [25]. An increase in cAMP levels resulting from constitutive Ras signalling has also been shown to lead to the inability of cells to regulate carbohydrate storage, oxidative phosphorylation, and stress responses during the stationary phase of growth [25,26]. Decreased cAMP/PKA activity can also lead to reduced growth and entry into G_0_ [24,27]. Ras2/cAMP/PKA signalling control in yeast is therefore crucial for the coordination of cell growth and an appropriate response to available nutrition.

We sought to further our knowledge of Ras2 regulation by identifying novel phosphorylation events that are important for its localisation. Our initial findings suggested that serine 225, which may be regulated by Ssn3 and Sit4, represents a putative candidate. However, as we observed the same phenotypes when we overexpressed either *RAS2^S225A^* (non-phosphorylatable) or *RAS2^S225E^* (phospho-mimetic), our findings do not strictly confirm that phosphorylation/dephosphorylation at serine 225 is responsible for directing localisation. The overexpression of either *RAS2^S225A^* or *RAS2^S225E^* did lead to mislocalisation to the nuclear envelope, which may suggest perturbation of processing the CAAX motif that is required for the transit of Ras2 to the plasma membrane [6]. Alternatively, this aberrant localisation may involve its interaction with the Ras inhibitor 1 (Eri1), which has been reported to enable contact of GTP-bound Ras2 with the ER via a role within the ER-localised GPI-GnT complex [11,12]. Mislocalised *RAS2^S225A^* or *RAS2^S225E^* was also found to be constitutively active, suggesting that GAP proteins were either not present or insufficient to regulate GDP/GTP activity under the conditions and expression levels used. Similar observations of constitutive activation have been reported under conditions or genetic backgrounds where Ras2 has been found to be localised to the mitochondrial compartment [14]; however, in these studies, this led to cell death consistent with yeast apoptosis. Overall, this adds to the evidence that the location and activity state of Ras2 are crucial in determining cell fate in yeast. The loss of viability and phenotypes attributed to overexpression and mislocalisation of either *RAS2^S225A^* or *RAS2^S225E^* could be attributed to enhanced cAMP/PKA activity. However, these effects were only observed above a threshold level of expression, suggesting that *S. cerevisiae* possess significant capacity for Ras regulation. It is tempting to speculate that a similarly high threshold exists in human cells, as RAS gene duplication is common in the manifestation of a number of cancers [28]. 

The loss in viability observed upon the overexpression of *RAS2^S225A^* or *RAS2^S225E^* was accompanied by an inability to re-engage in cell division upon the addition of fresh medium. One possibility is that the overexpression of *RAS2^S225A^* or *RAS2^S225E^* led to the dysregulation of essential processes required for growth. In line with this, we observed upregulation of some pathways required to support growth, such as mRNA translation, alongside simultaneous downregulation of others, such as DNA replication and glucose metabolism. The effects of *RAS2^S225A^* or *RAS2^S225E^* in coordinating cell-cycle entry could be overcome by the addition of peptone or the deletion of *CUP9*, which, in addition to leading to an increase in the expression of the di/tripeptide transporter *PTR2* [29], also led to the upregulation of a range of metabolite transporters. This suggests that Cup9 may be important in the suppression of a range of metabolic process and requires further investigation. One explanation for this finding is that the deletion of *CUP9* allows *RAS2^S225A^*- or *RAS2^S225E^*-overexpressing cells trapped within G_0_ following nutrient depletion to overcome an essential metabolic checkpoint that is required for cell-cycle re-entry. In yeast, a range of metabolites are known to influence progression through each stage of the cell cycle, in what has been termed the yeast metabolic cycle [30]. However, to the best of our knowledge, this has not been linked to Cup9’s function to date. 

Clearly, the quiescence observed in *RAS2^S225A^*- or *RAS2^S225E^*-overexpressing cells arises as a combination of nutrient limitation and aberrant Ras signalling. These findings highlight the crucial interplay among environmental adaption, signalling, and cell fate determination that is coordinated by Ras2. This is an emerging area of study that holds promise in the field of cancer research, where new approaches to manipulate metabolic checkpoints are being pursued as options for developing new therapies [31], and also in antifungal development. Fungal-specific domains that exist outside of the highly conserved GTPase domain have been reported and may be targeted to develop new antifungal therapies to combat human fungal pathogens such as *Aspergillus fumigatus* [32] and *Candida albicans* [33]. A recent study identified a synthetic peptide that can prevent Ras from interacting with its GEF, Cdc25, at a *C. albicans*-specific domain in the Ras1 protein [33]. As this interaction was shown to be important in regulating the yeast-to-hyphal switch, a key determinant of virulence in *C. albicans*, this represents an exciting new potential route to Ras-targeted infection control. Overall, the recognition that Ras signalling can be manipulated to reliably alter cell fate outside of the “undrugable” GTPase domain [17] is an attractive approach to developing new methods to control its activity.

## Data Availability

Data is available at Mendeley Data, V1, doi: 10.17632/pswx38b2zb.1 or upon request.

## Supplementary Materials

The following supporting information can be downloaded at: https://www.mdpi.com/article/10.3390/biom13111619/s1. Figure S1: Active Ras localisation in kinase a phosphatase deletions that lead to changes in phosphoryaltion status of Ras2; Figure S2: Western blot showing the detection of Ras2 in cells overexpressing *RAS2, RAS2^S225A^, RAS2^S225E^* from a low copy plasmid; Figure S3: Western blot showing the detection of Ras2 in cells overexpressing *RAS2, RAS2^S225A^, RAS2^S225E^* from a high copy plasmid; Figure S4: Western blot showing the detection of GFP in GFP-ATG8 cells overexpressing *RAS2*, *RAS2^S225A^*, *RAS2^S225E^*; Figure S5: Colony forming unit assay in cells overexpressing *RAS2*, *RAS2^S225A^*, *RAS2^S225E^* with additional glucose supplement; Table S1: Phospho pep 2.0 output for kinases and phosphatsases whose deletion led to a significant change in Ras2 peptide phosphorylation levels as detected by mass spectrometry; Table S2: Gene Ontology analysis of upregulated genes clustered to the transmembrane transporters function when transcriptomes of *RAS2^S225A^* and *Δcup9 RAS2^S225A^* cells were analysed; Table S3: Upregulated genes clustered to the GO Process term by Yeastmine when transcriptomes of wild type and Δcup9 cells were analysed; Table S4: Downregulated genes clustered to the GO Process term by Yeastmine when transcriptomes of wild type and Δcup9 cells were analysed.
