# Supplementary material for: Elevated Levels of Mislocalised, Constitutive Ras Signalling Can Drive Quiescence by Uncoupling Cell-Cycle Regulation from Metabolic Homeostasis
Source: Biomolecules. 2023 Nov 6;13(11):1619. doi: 10.3390/biom13111619 (PMC10669370; doi:10.3390/biom13111619)
Supplement: Supplementary file 1 [file biomolecules-13-01619-s001.zip › biomolecules-2688900-supplementary.pdf]

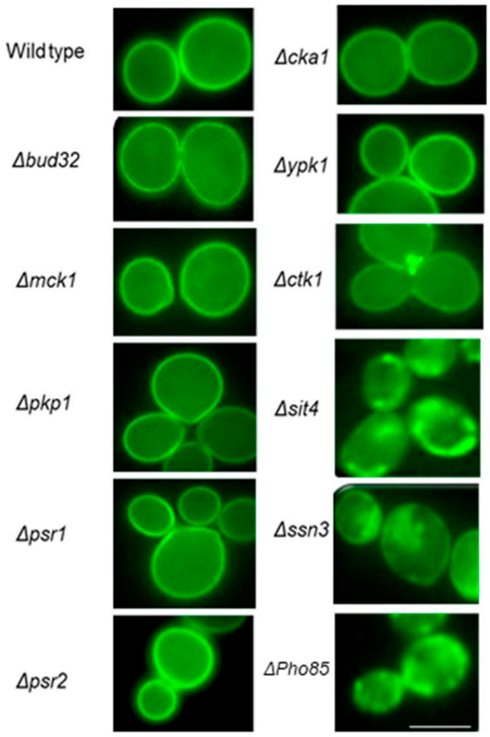

Figure S1 – Wild type cells or cells deleted for a single gene as indicated were transformed with a plasmid expressing the active Ras probe, 2 x RBD-GFP. The cells were cultured in SD-URA media containing 2% glusoce to select for the plasmid and sampled during log growth after inoculation. This experiment was repeated three times and a representative data set is shown. Size Bar = 5µm

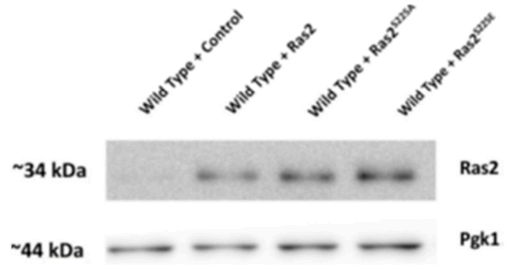

Figure S2 -A Western blot showing the detection of Ras2p in cells overexpressing *RAS2, RAS2^{S225A}, RAS2^{S225E}* or containing and empty plasmid control (low copy, CEN). The cells were cultured in SD-URA media and sampled during stationary phase of growth 24 hours after inoculation. Uniform protein loading was ensured by probing levels of Pgk1 after probing for Ras2p. This experiment was repeated three times and a representative data set is shown.

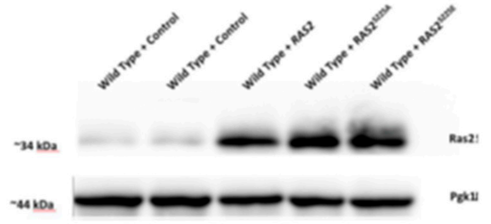

Figure S3 - A Western blot showing the detection of Ras2p in cells overexpressing *RAS2, RAS2^{S225A}, RAS2^{S225E}* or containing and empty plasmid control (high copy, 2µ). The cells were cultured in SD-URA media and sampled during stationary phase of growth 24 hours after inoculation. Uniform protein loading was ensured by probing levels of Pgk1 after probing for Ras2p. This experiment was repeated three times and a representative data set is shown.

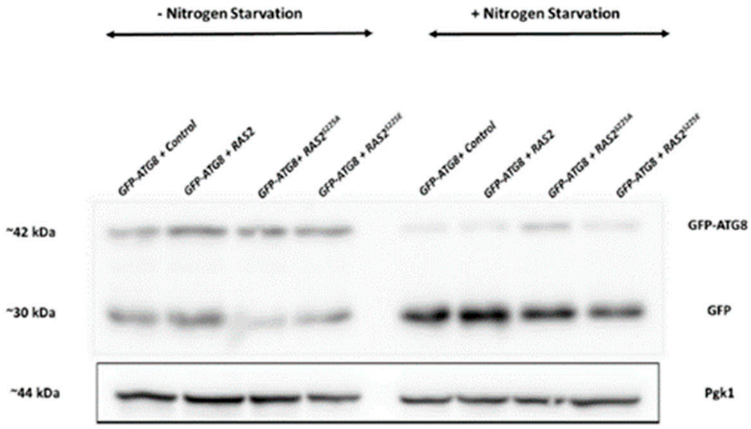

Figure S4 - A western blot showing the detection of GFP in GFP-ATG8 cells overexpressing RAS2, RAS2S225A, RAS2S225E or containing an empty plasmid control. Cells were grown in SD –URA media for 24 hours. 1ml of culture was transferred to nitrogen starvation media to induce autophagy via nitrogen starvation and incubated at 30°C with 200 RPM shaking for 6 hours. Total protein was extracted from cells pre- and post-induction of autophagy and subjected to western blotting. To ensure uniform protein loading, the levels of Pgk1p were detected after probing for Ras2p. This experiment was repeated three times and a representative data set is shown.

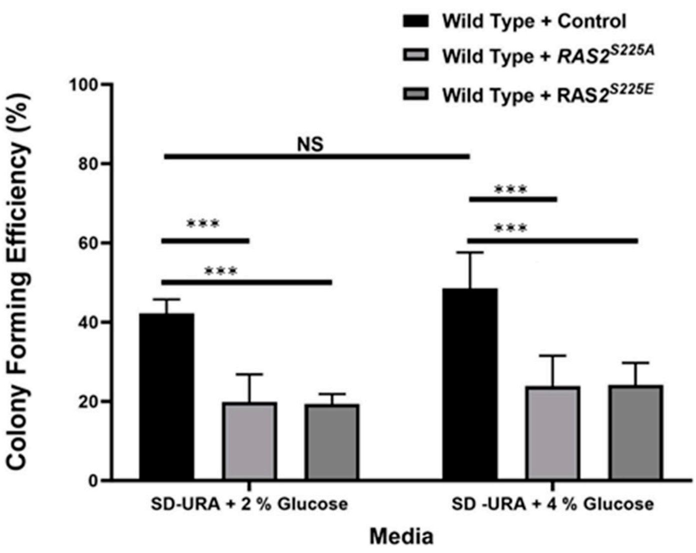

Figure S5 – Wild type cells or cells overexpressing *RAS2*^{S225A} or *RAS2*^{S2255} were cultured in SD-URA media containing wither 2% or 4% glucose for 24 hours and assessed for colony forming efficiency as described within materials and methods. This experiment was repeated three times and error bars represent standard deviation. A One-way ANOVA using a Tukey multiple comparison test was used to determine statistical significance. Non-significant = NS, *** = adjusted p value ≤ 0.001

Enzyme	Description	Ras2p phospho-peptide level in deletion strain	Identified sequence
YLR019W (PSR2)	Plasma membrane phosphatase, involved in general stress response	3.9 fold UP	R.KMS*NAANGK.N
YAR019C (CDC15)	Protein kinase of the mitotic exit network	1.7 fold UP	R.KMS*NAANGK.N
YGR262C (BUD32)	Protein kinase, required for tRNA modification	Down 2.2	R.KMS*NAANGK.N
YIL035C (CKA1)	Protein kinase, has a role in cell growth and proliferation	Down 2.2	R.NAS*IES* KTGLAGNQATNGK.T
YIL042C (PKP1)	Mitochondrial protein kinase, involved in negative regulation of pyruvate dehydrogenase	Down 1.5	R.NAS*IES* KTGLAGNQATNGK.T
YLL010C (PSR1)	Plasma membrane phosphatase, involved in general stress response	Down 3.1	R.NAS*IES* KTGLAGNQATNGK.T
YNL307C (MCK1)	Protein kinase, involved in chromosome segregation, mitotic entry, genome stability	Down 1.6	R.NAS*IES* KTGLAGNQATNGK.T
YPL042C (SSN3)	Cycline dependant protein kinase, involved in phosphorylation of RNA polymerase, and glucose repression	1.5 fold UP	K.GSGANSVPRNS* GGHR.K
YDL047W (SIT4)	Phosphatase, required for golgi to ER traffic, similar to human PP6	1.6 fold UP	K.NVNSS* TTVVNAR.N
YKL126W (YPK1)	Protein kinase; phosphorylates flippase activator Fpk1p, homolog of mammalian kinase SGK	1.6 fold UP	K.NVNSS* TTVVNAR.N
YKL139W (CTK1)	Catalytic subunit of C terminal domain kinase, similar to human CDK12	1.7 fold UP	K.NVNSS* TTVVNAR.N
YPL042C (SSN3)	Cycline dependant protein kinase, involved in phosphorylation of RNA polymerase, glucose repression	2.4 fold UP	K.NVNSS* TTVVNAR.N
YPL031C (PHO85)	Cyclin-dependent kinase, involved in cellular response to nutrient levels and environmental conditions and cell cycle	1.6 fold UP	R.NASIES* KTGLAGNQA

Table S1 – Phospho pep 2.0 output for kinases and phosphatsases whose deletion led to a significant change in Ras2 peptide phosphorylation levels as detected by mass spectrometry (ref)

ORF	Gene	Yeastmine GO term
YAL067C	SEO1	transmembrane transporter activity
YBL030C	PET9	transmembrane transport
YBL042C	FUI1	transmembrane transporter activity
YBL042C	FUI1	transmembrane transporter activity
YBR021W	FUR4	transmembrane transporter activity
YBR068C	BAP2	amino acid transmembrane transport
YBR069C	TAT1	amino acid transmembrane transport
YCL038C	ATG22	amino acid transmembrane export from vacuole
YCR023C		transmembrane transporter activity
YCR098C	GIT1	glycerophosphodiester transmembrane transport
YDL247W	MPH2	transmembrane transporter activity
YDR046C	BAP3	amino acid transmembrane transport
YER053C	PIC2	copper ion transmembrane transport
YER056C	FCY2	transmembrane transporter activity
YGL008C	PMA1	proton export across plasma membrane
YGR281W	YOR1	cadmium ion transmembrane transport
YGR289C	MAL11	carbohydrate transmembrane transport
YHL040C	ARN1	iron ion transmembrane transport
YHL047C	ARN2	iron ion transmembrane transport
YHR051W	COX6	proton transmembrane transport
YIL013C	PDR11	transmembrane transport
YJR095W	SFC1	succinate transmembrane transport
YKR093W	PTR2	dipeptide import across plasma membrane
YLL048C	YBT1	transmembrane transport
YLL061W	MMP1	amino acid transmembrane transport
YLR004C	THI73	transmembrane transporter activity
YLR038C	COX12	proton transmembrane transport
YLR152C		transmembrane transport
YLR214W	FRE1	iron ion transmembrane transport
YLR220W	CCC1	iron ion transmembrane transport
YMR241W	YHM2	mitochondrial citrate transmembrane transport
YNR055C	HOL1	transmembrane transporter activity
YOL152W	FRE7	iron ion transmembrane transport
YOR192C	THI72	5-amino-1-ribofuranosylimidazole-4-carboxamide transmembra
YPL058C	PDR12	organic acid transmembrane transport
YPL078C	ATP4	proton transmembrane transport
YPL092W	SSU1	transmembrane transport
YPL221W	FLC1	FAD transmembrane transport
YPL265W	DIP5	amino acid transmembrane transport
YPL274W	SAM3	amino acid transmembrane transport
YPR156C	TPO3	spermine transmembrane transport

Table S2 – Upregulated genes clustered to the transmembrane transporters function when transcriptomes of *RAS2*^{S225A} and *Δcup9 RAS2*^{S225A} cells were analysed by Yeastmine GO, P<0.0001

GO term	Enrichment P value	Genes within GO term
cytoplasmic translation	6.74E-08	RPL19B RPL23A RPS14A RPL31A RPP1B RPL41B RPL41A RPS18A RPS24A RPL30 RPL24A RPL1B RPL9A RPL26B RPL11B RPL42B RPL34B RPL43B TMA19 TMA7 RPS28B RPS30A RPS25B RPS18B RPL9B
pyruvate metabolism	2.39E-05	ENO1 ENO2 ERR3 FBA1 GPM1 IRC7 PDA1 PDA1 PDC1 PGI1 PGK1 TDH1 TDH2 TDH3 TPI1
glycolysis + carbohydrate metabolism	3.80E-05	PGI1 PGM2 PGK1 PDA1 MAL32 MAL12 PGK1 TPI1 EXG2 PDA1 TDH1 TDH2 TDH3 ENO1 GUT1 ENO1 ENO2 GND1 YHR210C IMA1 IMA2 IMA5 GPM1 GPH1 FBA1 ERR3
organic acid metabolism	6.23E-04	ETR1 PGI1 ARO4 HIS4 PGK1 ELO2 GLT1 LYS20 GCV1 TPI1 HOM2 ARG82 GCN4 HPA3 CEM1 PDA1 IRC7 GUS1 ADE6 VAS1 TDH3 ENO1 YHR020W ENO2 GND1 TDH1 URA2 TDH2 FBA1 GPM1 PDC1 SHM2 ASP3-3 ILV5 CYB2 ALD3 GCV2 GAD1 SNO4 ERR3 PYP1 LEU4 WRS1 LEU9 ORT1 GDH1 HSP33 HSP32 MRI1

Table S3 – Upregulated genes clustered to the GO Process term by Yeastmine when transcriptomes of wild type and *Δcup9* cells were analysed

GO term	Enrichment P value	Genes within GO term
rRNA modification	<0.05	BUD23 SNR82 SNR39B SNR32 SNR71 SNR3 SNR54 SNR76 SNR83 SNR86 SNR49 SNR81 SNR51 SNR70
Sulphur metabolism	<0.05	CYS3 MET8 MET10 MET3 MET14 MET16

Table S4 – Downregulated genes clustered to the GO Process term by Yeastmine when transcriptomes of wild type and *Δcup9* cells were analysed
